# Surgical Treatment of Persistent Fetal Vasculature and Visual Rehabilitation: One-Year Followup

**DOI:** 10.1155/2012/687081

**Published:** 2012-02-06

**Authors:** N. Kozeis, K. T. Tsaousis, D. Gidaris

**Affiliations:** ^1^Department of Ophthalmology, Hippokration General Hospital, 54642 Thessaloniki, Greece; ^2^1st Department of Paediatrics, Hippokration General Hospital, Aristotle University of Thessaloniki, 54642 Thessaloniki, Greece

## Abstract

We present the management and postoperative course of a persistent fetal vasculature (PFV) case. A four-year-old girl visited the Eye Department of Hippokration, General Hospital of Thessaloniki due to reduced visual acuity of her left eye. She was diagnosed with PFV and underwent surgery (lensectomy, capsulorhexis of the posterior capsule, insertion of an intraocular lens in the posterior chamber, and posterior vitrectomy) in order to dissect the PFV. Along with the postoperative medical care, she underwent intensive treatment for amblyopia. The postoperative course was uncomplicated, and the visual acuity of her left eye improved from hand movement to 20/25 with proper correction. Patients with unilateral PFV and gradually deteriorating visual acuity could be good candidates for a combined surgical procedure, as the one described above, with a good prognosis.

## 1. Introduction

Persistent fetal vasculature (PFV) is a congenital disease that usually appears unilaterally in otherwise normal children and can be associated with a smaller eye or a smaller cornea [1]. It is the most frequent cause of leukocoria, and it can be mistaken for retinoblastoma [2].

It is characterised by persistence and secondary fibrovascular proliferation of the primary fetal vasculature. The hyaloid system is prominent in the development of the eye. As the hyaloid artery regresses in normal development, the tissue reabsorbs. When a portion of retrolental posterior vasculosa lentis fails to resorb, it persists as an insignificant opacity on the posterior surface of lens (Mittendorf dot), and the disc shows an attached ghost artery. The vascular net can form a retrolental mass into which elongated ciliary processes are inserted. If the mass contracts the ciliary processes and the pupil is pulled centrally, the depth of the anterior chamber can be decreased causing secondary glaucoma. Contraction of the mass can also cause tractional retinal detachment [3, 4]. An associated dehiscence involving the posterior capsule may lead to subsequent cataract formation [5].

## 2. Case Report

**Figure 1:**
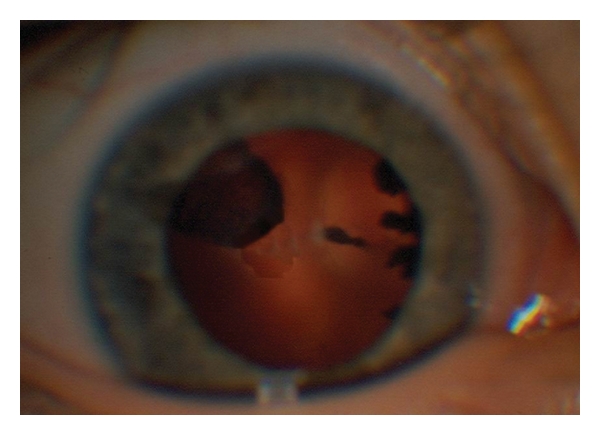
Persistent fetal vasculature (PFV).

A 4-year-old girl was referred to the ophthalmology department due to gradual reduction of the visual acuity of her left eye to hand movement. Her right eye visual acuity was 20/20 with proper correction (+2.00 sph, +0.25 cyl × 60°). Physical and mental development was within normal range. Her mother had an uncomplicated pregnancy and labour. On slit lamp examination, traction of the ciliary processes to the centre of the posterior capsule of the lens in her left eye and a retrolental mass were identified (Figure 1).

Biomicroscopy of her left eye with a 90-diopter lens revealed the presence of a posterior persistent fetal vasculature (fibrovascular tissue in Cloque's canal). No significant anatomical abnormalities in the vitreous base and peripapillary area were identified.

The eye was not microphthalmic (axial length: 22.46 mm and K1 = 43.50 dioptres, K2 = 43.16 dioptres) while intraocular pressure, measured with Perkins tonometer, was 14 mm Hg in both eyes. Right eye examination was unremarkable.

 It was decided that she should undergo surgery in order to remove the cataractous lens and the PFV. Under general anaesthesia, we performed aspiration of the crystalloid lens through a clear cornea incision, capsulorhexis of the posterior capsule, and removal of the retrolental mass and the ciliary processes with a vitreotome and insertion of an acrylic intraocular lens (IOL) at the sulcus. Then we proceeded to a pars plicata posterior vitrectomy and dissection of the posterior persistent fetal vasculature.

 On the first day after surgery, the patient's left eye had a clear cornea, the anterior chamber had no signs of inflammation, IOL was in situ, and retina was normal. Her left eye visual acuity was 20/200 without correction. She was advised to follow a treatment for amblyopia of her left eye (six-hour occlusion of her right eye daily). In addition she was prescribed topical antibiotic-steroid drops as well as drops of nonsteroid anti inflammatory eye drops for two months. Administration of the antibiotic-steroid drops was tapered appropriately.

 Three months after operation, visual acuity of her left eye was 20/50 with correction (−1.25 sph. −0.75 cyl. × 180°). She was advised to continue the treatment for amblyopia. Eight months later, visual acuity of her left eye further improved to 20/25 with correction (−0.75 sph. −0.75 cyl. × 180°).

## 3. Discussion

Once the retrolental mass (formed by the persistent fetal vasculature) does not cover the visual axis during the first year of life, the prognosis for patient's vision is excellent, provided that surgery and treatment for amblyopia of the affected eye will take place as soon as possible [5–7].

The anterior limbal approach selected for the cataract removal procedure, while pars plicata vitrectomy is preferred for the dissection of the fibrovascular tissue due to some degree of tractional stretching of ciliary processes and vitreous base.

 The combination of pars plicata vitrectomy, extraction of the cataractous lens, and insertion of an IOL at the same time had a satisfactory result for the patient's rehabilitation and showed no disadvantage compared with a vitrectomy and an IOL insertion in two separate surgeries [8, 9].

We used intraocular lens of hydrophobic acrylic material (Acrysof, Alcon, Fort Worth, TX), and no severe postoperative inflammation is expected. However, we recommended topical steroid for almost two months in order to avoid postoperative complications as peripapillary membranes and posterior capsular opacification.

 Prognosis is poor for neglected cases or for patients who develop secondary glaucoma or tractional retinal detachment due to contraction of the persistent fibrovascular net [10].
